# Survey dataset on reasons why companies decide to implement continuous improvement

**DOI:** 10.1016/j.dib.2019.104523

**Published:** 2019-09-16

**Authors:** Lidia Sanchez-Ruiz, Beatriz Blanco

**Affiliations:** University of Cantabria, Spain

**Keywords:** Continuous improvement, Kaizen, Motivation, Dataset

## Abstract

Continuous improvement practices are increasingly common among all kinds of companies as a means to achieve business excellence. However, the reasons why companies implement continuous improvement might vary a lot. This article presents data on the motivations that induce companies to develop continuous improvement initiatives. The data were collected through a survey. Of the 209 companies surveyed, 109 answered it. The dataset includes information about the main characteristics of the company (size, sector) together with information about the main reasons to set up continuous improvement management practices. The dataset is available in excel format. The authors consider that these data are useful not only for researchers but also for consultants and for those practitioners interested in decision sciences.

**Table:** Specifications Table

Subject area	*Business and management*
More specific subject area	*Process management and continuous improvement*
Type of data	*Table*
How data was acquired	*Survey*
Data format	*Raw data*
Experimental factors	*Survey was previously validated by consulting experts in continuous improvement initiatives such as full professors, company managers, consultants.* *Survey was conducted among companies that have implemented continuous improvement practices*
Experimental features	*Continuous improvement initiatives are increasingly spreading among all kind of companies . Thus, understanding why companies implement continuous improvement can help to develop better practices and improve the decision management process*
Data source location	*Cantabria (region in the North of Spain)*
Data accessibility	*Data are included in this article (Supplementary material)*

**Table:** 

**Value of the data** • The data can be useful to researchers to understand better the behavior of companies and to develop new theories about decision management and organizational behavior. • Not only might the dataset be helpful for researchers, but also for consultants. Understanding companies' motivations to implement continuous improvement will allow them to understand better companies' decisions, so advisors will be able to offer more specific and customized solutions and/or consulting services. • It is an indication of what really concerns companies: therefore, it might be useful for policy makers when promoting programs that encourage the implementation of continuous improvement. • It is also useful for the international organisms responsible for the development of international standards based on continuous improvement (ISO, EFQM …) to better understand the point of view of companies. • As a future line of work, studies could be conducted that classify the motivations according to their nature: Are there different types of motivations? Which ones predominate? Are motivations the same between sectors? What are the most important motivations? Additionally, subsequent studies could be developed to analyze whether the motivations influence the results. That is, is the failure rate the same or does it vary depending on the main motivation of the company? Do all companies obtain the same results regardless of their initial motivations?

## 1 Data

The dataset includes information on the main reasons why companies decide to implement continuous improvement (Kaizen) in Cantabria (northern region of Spain).

The survey (Appendix 1) included several general questions about the company itself (Questions 1 to 5) and a question about motivations to implement continuous improvement programmes (question 6). In this last question, companies had to assess each of the motivations on a scale of 1 (it was not an important reason for our company) to 5 (it was one of the main reasons for our company). The process of selecting the variables is explained in the following section.

**Fig. 1:**
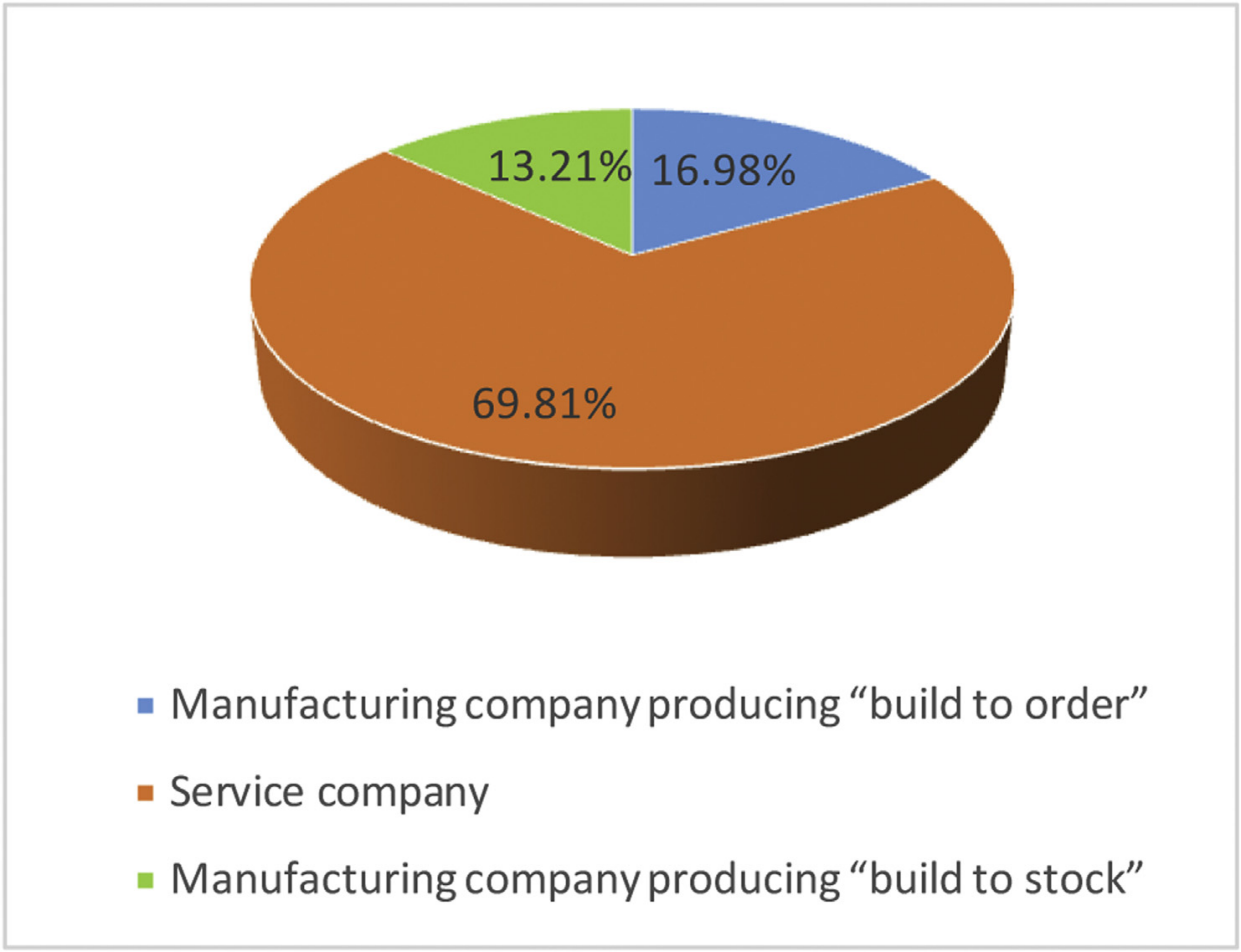
Respondents' distribution based on the kind of company.

**Fig. 2:**
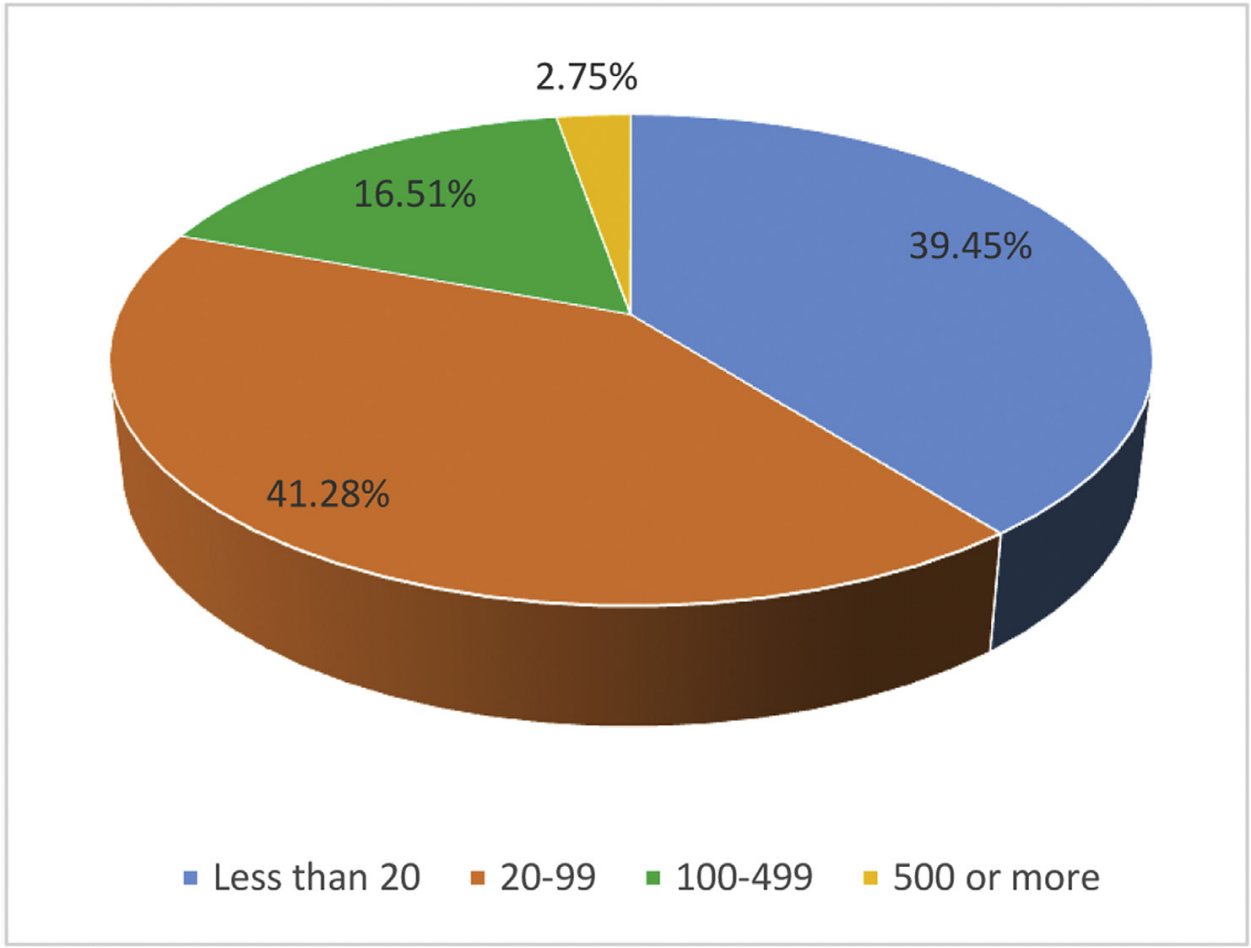
Respondents' size distribution (number of employees).

Finally, 109 valid answers were obtained (data collection process is explained in detail in the following section). Fig. 1 shows the distribution according to the type of company; Fig. 2 includes the distribution of companies according to their size. Table 1 shows the distribution by sector according to Spanish National Classification of Economic Activities and finally, Table 2 shows the frequency distribution for each of the motivations.

**Table 1:** Respondents’ sector distribution.

Spanish national classification of economic activities	Number of companies
C: Manufacturing	31
E: Water supply; sewerage, waste management and remediation activities	1
F: Construction	14
G: Wholesale and retail trade; Repair of motor vehicles and motorcycles	20
H: Transportation and storage	7
I: Accommodation and food service activities	2
J: Information and communication	1
K: Financial and insurance activities	2
M: Professional, scientific and technical activities	6
N: Administrative and support service activities	7
P: Education	2
Q: Human health and social work activities	10
R: Arts, entertainment and recreation	3
S: Other service activities	3
	109

**Table 2:** Respondents’ frequency distribution to continuous improvement motivations.

	1	2	3	4	5
Discovering what is happening in the company	16,5%	9,2%	17,4%	33,9%	22,9%
Customer pressure	39,4%	24,8%	22,0%	8,3%	5,5%
Auditing the company's culture	25,7%	15,6%	27,5%	23,9%	7,3%
Performance measurement	23,9%	13,8%	24,8%	22,0%	15,6%
Identifying improvement opportunities	9,2%	1,8%	11,9%	33,0%	44,0%
Supplier pressure	59,6%	18,3%	18,3%	1,8%	1,8%
Internal benchmarking	35,8%	21,1%	19,3%	16,5%	7,3%
As a part of a remuneration policy	57,8%	25,7%	10,1%	4,6%	1,8%
As a part of a wider system (Lean Management, ISO 9000 …)	16,5%	4,6%	12,8%	23,9%	42,2%
Discovering what customers want	15,0%	15,0%	18,7%	30,8%	20,6%

## 2 Experimental design, materials, and methods

The data was obtained through a survey. This was aimed at those people responsible for the implementation of continuous improvement. In order to develop this study, the first step consisted in the design of the survey, which, as already mentioned in the previous section, consisted of several identifying questions (sector, size, type of company) and a question about the motivations for implementing the continuous improvement.

The first step involved carrying out a bibliographic review of the literature that would allow the authors to identify the motivations that had been previously stated in the literature and that, therefore, had to be included in the survey.

The literature review was carried out in the Web of Science and Scopus databases, using the keywords "continuous improvement" and "kaizen" [1]. After reviewing and analyzing the papers which were about this topic, the main motivations for the implementation of continuous improvement programs were identified. Table 3 summarizes the main motivations identified in the aforementioned review.

**Table 3:** Main continuous improvement motivations based in the literature review.

	Middel et al. [2]	Albors Garrigós et al. [3]	Mejías Sacaluga et al. [4]	Terziovski and Sohal [5]	Fryer and Douglas [6]
Increasing customer satisfaction	X	X	X		
Increasing productivity	X	X	X	X	
Improving quality	X	X	X	X	
Increasing reliability and time delivery	X	X	X	X	
Cost reduction	X	X	X	X	
Improving management	X			X	
Improving cooperation	X		X	X	
Fostering innovation	X				
Improving communication	X		X	X	
Increasing staff commitment towards change	X	X	X	X	
Improving secutiry and safety working conditions	X	X	X		
Improving the relationship among functional departments	X	X	X		
Increasing the abilities and skills of the employees	X	X	X	X	
Reducing process times	X			X	
Increasing production volume	X	X		X	
Customer preassure	X	X		X	X
Improving relationship with suppliers	X	X			
Increasing flexibility			X		
Supplier preassure	X				X
Competition preassure	X				
Reducing absenteeism		X			
Management decision		X			

Since there was a large number of motivations, and with the aim of making a rigorous design of the survey, it was validated by consulting experts. These were professionals from academia (including Full Professors of Business Organization), business managers (both manufacturing and services), consultants and quality managers. After completing this process, the survey was finally composed of 10 items (See Appendix 1, Question 6). It should be noted that the validity and reliability of the questionnaire were also validated [7].

The population of the study would be made up of companies with more than 20 employees that practise continuous improvement in Cantabria, a region in the north of Spain. However, due to the fact of not having a database on the number of companies that practice continuous improvement, as pointed out by Albors and Hervás [8], it was decided to send a first survey to all the companies with more than 20 employees in Cantabria. In it, in addition to a series of identifying data, they were asked if they practiced continuous improvement. This first stage had a main objective: to identify the companies that practise continuous improvement which, therefore, will made up the population of our study; and to which the second, more extensive, survey on the motivations to implement continuous improvement would be directed.

Therefore, the first survey was sent to the 808 companies located in Cantabria with more than 20 employees. They were identified using official data sources [9] and they were sent the first survey by email.

Of the 808 companies, 299 responded: 90 of them said that they did not practise continuous improvement; whereas, 209 of them said that they practiced continuous improvement and, therefore, they were sent the second survey (See appendix 1).

The survey was mostly conducted by email. However, when the manager required it, face-to-face structured interviews were done.

Of the 209 companies, 109 responded. This sample, although improvable, must be taken into consideration. There are few studies in the area of continuous improvement that work with this number of data. It must be borne in mind that in this field of research, descriptive studies based on the case of a company or a small group of companies predominate. This, without a doubt, we consider that it is an added value for the publication.

In order to evaluate the representativeness of the obtained sample and taking into consideration that, as above-mentioned, the size of the population is unknown, three scenarios are analysed (Table 4):

• Scenario 1: all the companies that did not answer the first survey did practise continuous improvement.
• Scenario 2: all the companies that did not answer the first survey did not practise continuous improvement.
• Scenario 3: some of the companies that did not answer the first survey did practise continuous improvement. As a proxy, it is assumed that the percentage of companies that would practise continuous improvement is the same as the one in the obtained sample, this is 69.89% (209/299).

**Table 4:** Estimation of the population in each of the three assumed scenarios.

	Companies with more than 20 employees in Cantabria	Companies that practise continuous improvement (population of our study)	Companies that do not practise continuous improvement
Scenario 1	808	209 + 509 = 718	90
Scenario 2	808	209	90 + 509 = 599
Scenario 3	808	209 + 509*(209/299) = 565	90 + 509*(90/299) = 243

On the basis of the 808 companies, the minimum number of answers needed so that the sample is representative is calculated for each of the scenarios making the formula shown below (Table 5).

$$n=\;\frac{\left(\frac{z^{2}*p\;(1-p)}{e^{2}}\right)}{1+\;\left(\frac{z^{2}*p*(1-p)}{e^{2}*N}\right)}$$

Where:

**Table 5:** Estimation of the minimum sample needed in each of the three assumed scenarios.

	Population size	E = Error rate	Z = 1.65 (90% level of confidence)	P = percentage	Minimum sample needed
Scenario 1	718	10%	1.65	50%	62
Scenario 2	209	10%	1.65	50%	51
Scenario 3	565	10%	1.65	50%	60

N = Population size

e = Error rate = 10%,

z = 1.65 (90% level of confidence),

p = percentage = 50%

It might be seen that the sample size obtained (109) is higher than the minimum sample needed in any of the three scenarios (Table 5), even in scenario 1 which is the most conservative one. Therefore it might be concluded that the obtained sample is representative.

## Figures and Tables

**Table:** 

	1	2	3	4	5
Discovering what is happening in the company					
Customer pressure					
Auditing the company's culture					
Performance measurement					
Identifying improvement opportunities					
Supplier pressure					
Internal benchmarking					
As a part of a remuneration policy					
As a part of a wider system (Lean Management, ISO 9000 …)					
Discovering what customers want					
Others (indicate)					

## APPENDIX 1 SURVEY

1. Company name:
2. Does your company have any of the following certificates?
  ISO 9000/9001
  ISO 14001
  OSHAS 18000
  EFQM
  OTHERS:
3. Company sector
4. Which kind of company is yours predominantly?
  Manufacturing company producing “build to order”
  Manufacturing company producing “build to stock”
  Service company
5. Number of employees:
  Less than 20
  20–99
  100–499
  500 or more
6. What are the reasons you decided to implement continuous improvement? 5 (very important reason) to 1 (it does not influence us, it was not a reason).

## References

[1] Sanchez L., Blanco B. (2014). Three decades of continuous improvement. Total Qual. Manag. Bus. Excell..

[2] Middel R., Op De Weegh S., Gieskes J. (2007). Continuous improvement in The Netherlands: a survey-based study into current practices. Int. J. Technol. Manag..

[3] Albors Garrigós J., Hervás Oliver J.L., Segarra Oña M. del V. (2009). Análisis de las prácticas de mejora continua en España: barreras y facilitadores. Econ. Ind..

[4] Mejías Sacaluga A., García Arca J., Fernández González A., Carlos J., Prado P. (2009). Análisis de la situación en España de la implantación de la Mejora Continua a través de sistemas de participación del personal. 3rd Int. Conf. Ind. Eng. Ind. Manag., Barcelona.

[5] Terziovski M., Sohal A.S. (2000). The Adoption of Continuous Improvement and Innovation Strategies in Australian Manufacturing Firms. http://www.elsevier.com/locate/technovation.

[6] Fryer K.J., Antony J., Douglas A. (2007). Critical success factors of continuous improvement in the public sector. TQM Mag..

[7] Sánchez Ruiz L., Blanco B. (2016). Construct validity in Operations Management by using Rasch Measurement Theory. The case of the construct “motivation to implement continuous improvement”. Work. Pap. Oper. Manag..

[8] Albors J., Hervas J.L. (2007). CI practice in Spain: its role as a strategic tool for the firm. Empirical evidence from the CINet survey analysis. Int. J. Technol. Manag..

[9] de Cantabria G. (2012). Cantabrian Institute of Statistics. https://www.icane.es/companies-directory.

